# Pilot training for clinical research professionals in using empathy to recognize and respond to implicit bias in research recruitment and retention

**DOI:** 10.1017/cts.2024.618

**Published:** 2024-11-18

**Authors:** Jennifer Adams, Cristina M. Gonzalez, Colleen Gillespie, James Holahan, Maura Minsky, Suchismita Datta, Rosario Medina, Amin Yakubov, Kimberly Byrnes, Miriam A. Bredella

**Affiliations:** 1 Center for Empathy in Medicine/ The Empathy Project, Institute for Innovation in Medical Education, NYU Grossman School of Medicine, New York, NY, USA; 2 Institute for Excellence in Health Equity and Departments of Medicine and Population Health, NYU Grossman School of Medicine, New York, NY, USA; 3 NYU Clinical and Translation Science Institute, NYU Langone Health and NYU Grossman School of Medicine, New York, NY, USA; 4 Department of Emergency Medicine, NYU Langone Hospital-Long Island and NYU Grossman Long Island School of Medicine, Mineola, NY, USA; 5 Department of Radiology, NYU Langone Health and New York University Grossman School of Medicine, New York, NY, USA

**Keywords:** Clinical research professionals, recruitment, patient engagement, implicit bias, empathy

## Abstract

Recruiting and retaining research participants is challenging because it often requires overcoming structural barriers and addressing how histories of mistrust and individuals’ lived experiences affect their research engagement. We describe a pilot workshop designed to educate clinical research professionals on using empathy skills to recognize and mitigate bias to improve recruitment and retention. In a post-workshop survey (22/31 participants completed), 94% agreed the workshop helped them practice perspective-taking, recognize implicit bias, and identify opportunities for empathy. Participants reported increased confidence in key recruitment and retention skills (*p* < 0.05). Future studies will evaluate whether this translates into improved recruitment.

## Introduction

Successful engagement, recruitment, and retention of diverse research participants is essential to achieving health equity. Yet, many studies fail to meet recruitment and retention goals [1]. Barriers to research participation are generally recognized to be even greater for individuals from marginalized communities – racial, ethnic, and other groups that face current structural inequities, including bias and discrimination, and for whom past events have led to mistrust in research and/or the healthcare system. This has led to disparities such that those disproportionately impacted with the greatest burden of disease are the least proportionally represented in research [2]. While changes are needed at all levels to eliminate these disparities, one practical approach is to empower clinical research professionals (CRPs) responsible for enrollment, recruitment, and retention with the skills needed to address these barriers.

Clinical research as a field has come to appreciate that participant engagement requires a complex set of sophisticated skills in order to build rapport, establish trust, explain and educate individuals about research, identify potential participants’ underlying concerns, and determine how to overcome practical barriers to participation [3]. Professionalizing this essential workforce by supporting professional development and advancement has become a high priority [4] and collaborative efforts have led to the development of competency frameworks for clinical research professionals that define skills necessary for effectiveness [5].

Relevant competencies include the specific skills needed to engage participants from under-represented, vulnerable, and/or minoritized patient populations. While early efforts in this area focused on trainings in “cultural competence” –defined as “the ability to engage knowledgeably with people across cultures” [6], this approach has had limited efficacy [7,8] and has been criticized for having the potential to reinforce stereotypes, disregard individual differences, downplay intersectionality, and assume that one person can know everything that should be known about all “cultures.” This has led to an appreciation for the concept of cultural humility/sensitivity as a “lifelong process of self-reflection and ability to recognize one’s biases and being open to and curious about patient experiences” [6]. This framework assumes that being culturally sensitive is a process, that people have multiple and intersecting and varying identities that can and do change, and that achieving cultural humility is based on a constantly developing, dynamic set of skills.

One area of focus within this framework is implicit bias – the unconscious and unintentional mental associations that impact our understanding and actions [9]. Implicit racial bias has been associated with negative clinician interactions with patients [10], less effective patient education [11], and decreased adherence to treatment plans [12]. Implicit bias has also been shown to negatively affect recruitment [13]. Implicit bias recognition and management (IBRM) is a patient-informed framework for learners to recognize when implicit bias is negatively influencing an encounter and then implement skills to manage that negative influence and optimize outcomes. IBRM skills include apologizing, understanding perspective, and checking in to restore rapport [14,15]. Empathy, defined broadly as the practice of authentically trying to understand another person’s lived experience through communication, and, more specifically, through engaged curiosity [16], has been shown to be an important ingredient in effective patient care [17]. Moreover, the skills of empathy – active listening, perspective-taking, and explicit acknowledgment of emotions and experiences – synergistically align with IBRM strategies and provide a promising approach to achieving equitable outcomes.

To address the limitations of prior cultural competency approaches in CRP professional development, we sought to explore whether an innovative curriculum designed for clinicians to use empathy as a core skill to recognize and respond to implicit bias could be adapted for and acceptable to CRPs. In this brief report, we describe an empathy workshop for CRPs and provide evaluation data on a pilot implementation of this workshop with three Clinical Research Centers of the Clinical and Translational Science Institute (CTSI) within our healthcare system.

## Materials and methods

The workshop was delivered to 31 Clinical Research Coordinators, Clinical Research Nurses, and Administrators at three Clinical Research Centers within the NYU CTSI.

### Curriculum description

The curriculum is grounded in a high-quality, animated film, The Elephant in the Waiting Room (https://www.empathyproject.com/denise), and captures the power of behavior observation [18] as an educational strategy. The film’s script was created with input from patients, healthcare professionals, and learners with the aim of creating a compelling, engaging, and realistic experience that would trigger meaningful discussion, reflection, and motivation for behavior change. The 7-minute film portrays a clinical encounter between a young, Black woman (Mariam Ouologuem) and a White male physician (Oliver Gunderson). The physician’s missteps based on his life experiences and the patient’s prior experience with discrimination in healthcare yield multiple opportunities for the use of empathy to restore rapport. Just as the encounter starts to fall apart, Denise the Empathy Elephant appears and coaches the physician to use core communication, empathy, and IBRM to recognize his biases, mitigate their impact, reconnect with the patient, and identify that she’s been misdiagnosed.

The 90-minute workshop (see detailed facilitator’s guide, **Appendix**) starts with a foundational “mini-lecture” that defines empathy and implicit bias, cites evidence on the impact of implicit bias, introduces the IBRM framework, and highlights the use of empathy as an IBRM skill and then engages participants in three active learning sections: 1) Reflection on the power of lived experience in shaping our biases; 2) Behavior identification and perspective-taking (and believing); and 3) Skills-building. The film is paused for teaching, discussion, and reflection points. We adapted this workshop for CRPs by incorporating the challenges of recruitment into the initial presentation, exploring the similarities and differences between the provider/patient and the CRP/research participant relationship, and addressing the ways in which implicit bias might influence patient engagement throughout the discussion, reflection, and group debrief.

### Evaluation

We focused on three early implementation questions: 1) How did the participants perceive the usefulness of the experience for their work as CRPs? 2) What is the impact of participating on CRPs confidence to perform core recruitment and retention tasks? 3) How could this workshop be improved and/or supplemented to maximize its effectiveness?

Participants were asked to complete a relatively brief anonymous online survey (via Qualtrics). The survey collected basic demographic and prior training information and elicited participants’ views on the workshop and its likely impact using a 4-point Likert-type scale (Strongly Disagree, Somewhat Disagree, Somewhat Agree, Strongly Agree). Items were adapted from prior evaluation surveys used with more than 300 participants in the clinical care-focused version of the workshop. Participants also retrospectively rated their pre-workshop confidence in performing twelve recruitment and retention skills and then their post-workshop confidence and the significance of differences was analyzed with paired t-tests. This pre-post retrospective design attempts to correct for the tendency of participants in brief training programs to overestimate their confidence in targeted skills if asked *prior* to the training and then provide a more accurate estimation *after* learning more from the training [19]. Items were developed through a review of the literature and were designed to reflect specific, discrete skills essential to recruitment and retention. Open-ended questions invited participants to share their thoughts on what worked well and what could be improved. Finally, we emailed participants three months after participation and asked them how well they remembered the workshop, whether they had applied anything they had learned from the workshop in practice, and thoughts on how best to build on and reinforce the goals of the workshop.

This project was designed as a quality improvement/program evaluation project and, per our IRB’s self-certification process, did not require human subjects review.

Statistical analyses were conducted using SPSS (IBM, Released 2021) [20]. Descriptive statistics were calculated for workshop participant demographics and frequency distributions were provided for prior trainings, views on the workshop, and post-workshop feedback. Paired t-tests were used to compare self-reported pre-workshop with post-workshop competencies (two-sided *p* values provided).

## Results

Twenty-two out of 31 participants (71%) completed the evaluation survey (response rates at each site were 85%, 66%, and 58%, respectively).

Demographics and prior trainings of research participants are shown in Table 1. While most participants had prior training in informed consent and recruitment/retention, fewer reported training that focused on the more specific skills of incorporating shared decision-making, handling disruptive behavior, and engaging with non-English speaking participants.

**Table 1.** Participant characteristics and prior training (*n* = 22)

	Percent (%)	N
**DEMOGRAPHICS**		
Gender		
Women	59%	13
Men	27%	6
Other or Prefer Not to Say	14%	3
Race/Ethnicity (multiple responses)		
White	33%	8
Asian	29%	7
Hispanic/Latino, a	8%	2
African-American/Black	8%	2
Other or Prefer Not to Say	21%	5
**PRIOR TRAININGS PARTICIPATED IN**		
Informed consent training for participants	68%	15
Social determinants of health training (how to recognize social determinants of health that may impact study participation)	50%	11
Training on how to effectively communicate with patients during the recruitment process	50%	11
Training on the history of research in the U.S. that may lead some individuals to be hesitant to become involved in research	50%	11
Training focused on recruitment and retention of minority (under-represented in research) populations	50%	11
Cultural competence/humility training (best practices for engaging with potential research participants in ways sensitive to their lived experiences/backgrounds)	41%	9
Training to elicit and address patients’ concerns about research (e.g., mistrust, fear, etc)	38%	8
Training on the specific skills needed to share decision-making with patients as part of the informed consent process	27%	6
Disruptive participant training (how to respond to research participant’s inappropriate or unruly behavior)	23%	5
Language and/or interpreter training (best practices for engaging with non-English speaking participants)	18%	4

**Figure 1.**
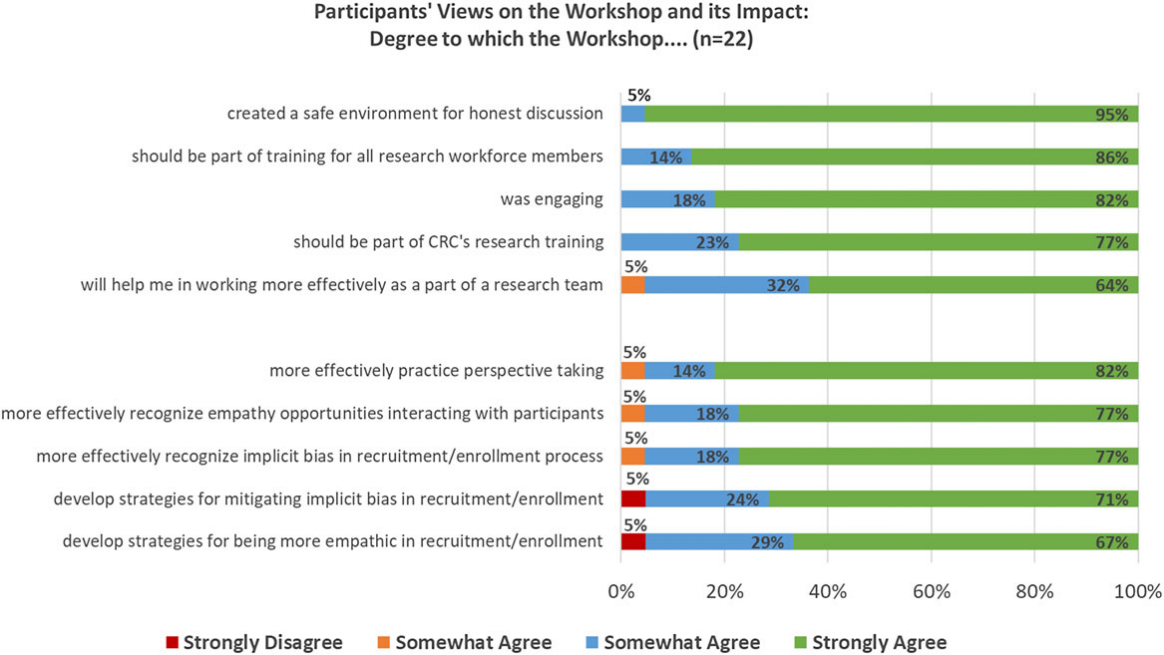
Participants’ views on the workshop and its impact (*n* = 22).

All of the participants rated the workshop experience positively (agreeing that it was engaging, should be part of training, and provided a safe environment) (Figure 1). Slightly fewer, but still almost all, felt that the workshop would help them in their work (e.g., being more empathic, recognizing and addressing implicit bias, working as part of a research team).

**Figure 2.**
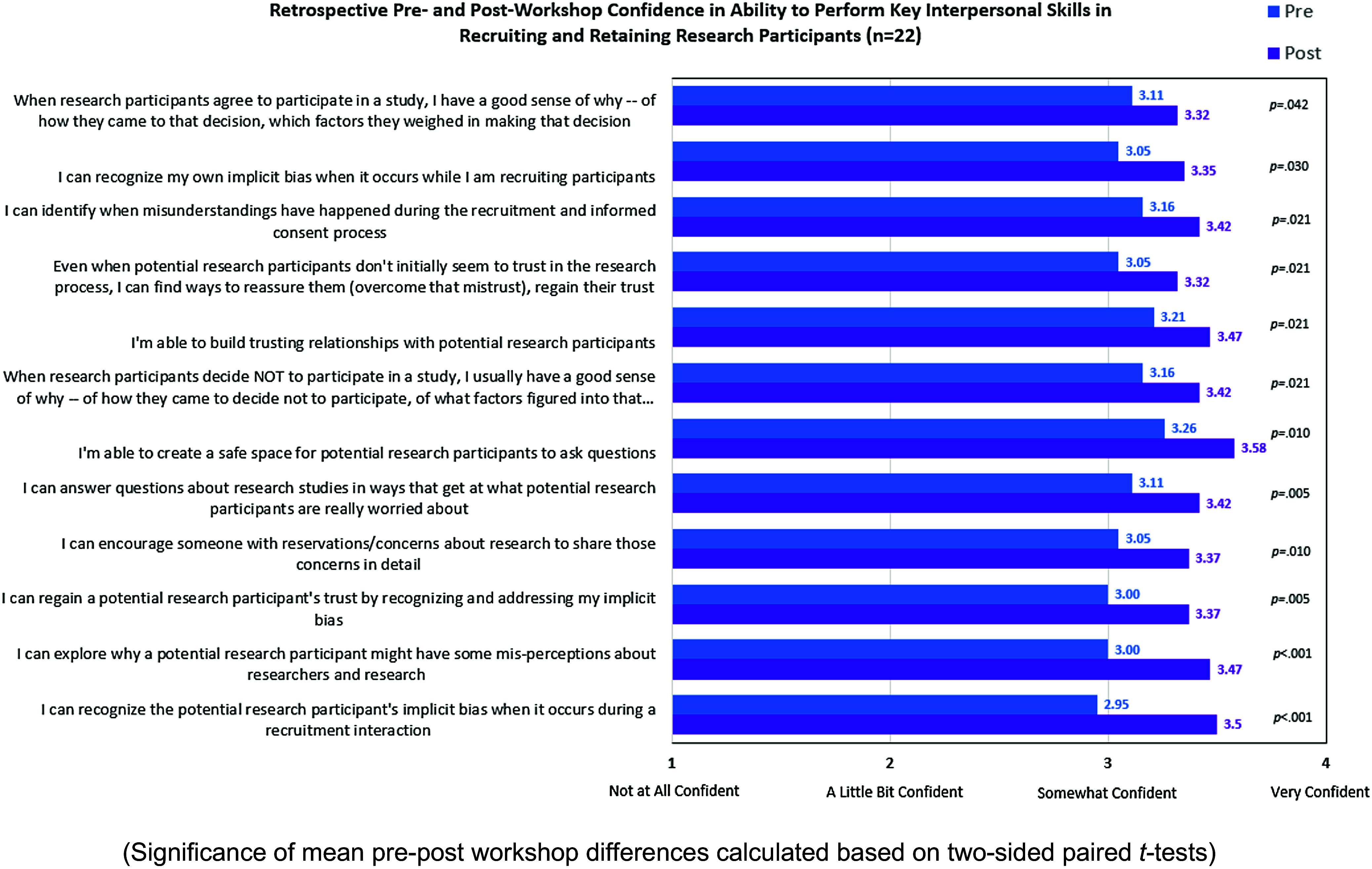
Self-reported change in confidence in ability to perform skills (Retrospective pre- vs post-workshop) (*n* = 22).

Participants’ confidence in their ability to perform skills particularly relevant to recruitment and retention of under-represented populations (Figure 2) was significantly greater after the workshop than before (p < 0.05) with effect sizes > 0.50 (Cohen’s D) for all twelve items. The greatest increases in confidence were seen for regaining a research participant’s trust by recognizing and addressing one’s own implicit bias, exploring misperceptions about research, and recognizing a research participant’s implicit bias.

In open-ended questions participants had positive comments on the workshop, highlighting the engaging nature of the interactive sessions and noted the power of the film. They also provided constructive suggestions for improvement, suggesting more focus on the specific challenges of recruitment and retention, greater attention to skills-building and tools, and expanding the focus to include ageism, generational assumptions, sexism, language barriers, education differences, and economic disparities.

In the brief follow-up survey of participants three months after the workshop, 11/22 responded and reported they remembered the workshop, “extremely well” (6/11) and “moderately well” (5/11), and provided examples of application in practice. Participants suggested more skills-building sessions with real-life examples as next steps.

## Discussion

Our workshop on using empathy to recognize and respond to implicit bias in recruitment and retention of research participants was well received by clinical research coordinators, research nurses, and research administrators from three Clinical Research Centers of the NYU CTSI, suggesting that such trainings could be incorporated into professional development activities for CRPs. Our findings of significant increases in confidence in tackling the specific skills of recruiting and retaining minoritized patients and retention bodes well for the ways in which this workshop (and others like it) could improve engagement across a range of patients. Participants also made clear, however, that they thought that the workshop should be reinforced with subsequent focus on more research-specific examples and opportunities to develop and practice the actual skills of using empathy to recognize and manage implicit bias.

Following the lead of the competency-based movement in medical education, we sought to identify core skills in recruitment and retention that can generalize across research engagement tasks and are both relevant to all patient populations and essential for minoritized individuals. As we have found in our clinically-focused version of the workshop, empathy, and IBRM appear to provide a useful framework for defining strategies for addressing implicit bias. We plan to implement experiential training to further build and reinforce these skills and to investigate whether these skill enhancements are linked to more effective research engagement. If we find evidence of impact, we would recommend that competency frameworks for CRPs [5] be expanded to include these skills.

This exploratory study has many limitations, principally a small sample from one large urban CTSI in the Northeast with self-reported, short-term outcomes, but provides an initial perspective on the potential usefulness of the next generation of targeted trainings for CRPs. Future studies should include suburban or rural CTSAs to validate our results. Moreover, different geographic or cultural contexts will likely require different case scenarios. Another limitation is that our scenario describes an interaction between a patient and a physician and might be stronger if it were between a research participant and a recruiter. We therefore plan to design and implement experiential trainings in these skills using simulation (exercises involving standardized patients playing the part of research participants where CRPs can practice and receive feedback), working with CRPs across our CTSI to create real-world scenarios that reflect the challenges to research likely to have the greatest impact and that can be transferred to CTSA with different populations. Further research can then explore the longer-term impact of these efforts, both on the effectiveness of recruitment and retention and on the careers and flourishing of CPRs themselves.

## Supporting information

Adams et al. supplementary material Adams et al. supplementary material

## Supplementary material

The supplementary material for this article can be found at https://doi.org/10.1017/cts.2024.618.
