# Supplementary material for: Pilot training for clinical research professionals in using empathy to recognize and respond to implicit bias in research recruitment and retention
Source: J Clin Transl Sci. 2024 Nov 18;8(1):e209. doi: 10.1017/cts.2024.618 (PMC11713438; doi:10.1017/cts.2024.618)
Supplement: Adams et al. supplementary material [file S2059866124006186sup001.pdf]

Working Towards Health Equity:

The Interplay Between Empathy and Implicit Bias

THE EMPATHY PROJECT
NYU Langone Health

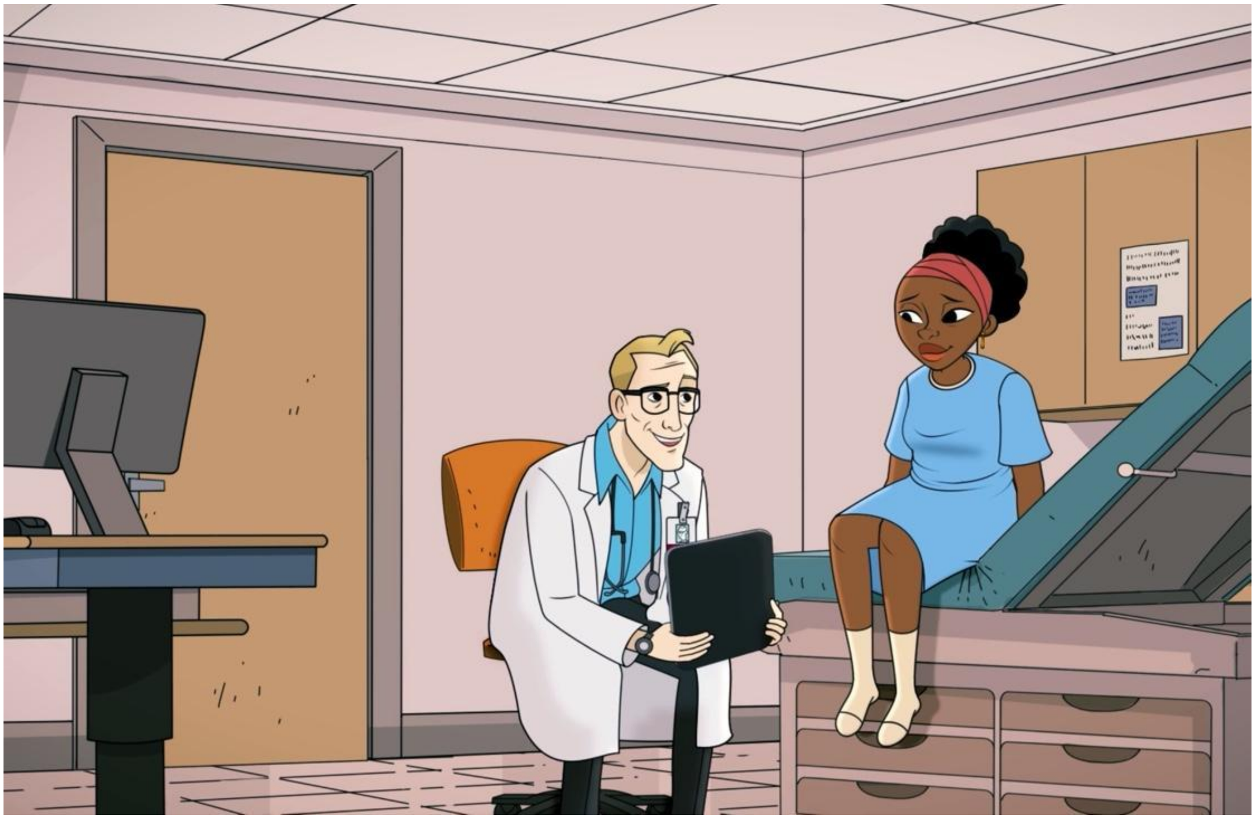

Working Towards Health Equity: The Interplay Between Empathy and Implicit Bias

Introduction

The Empathy Project (empathyproject.com), a nonprofit organization based within the NYU Grossman School of Medicine, created the short film, "The Elephant in the Waiting Room."

"The Elephant in the Waiting Room" is a 7-minute animated film that addresses implicit bias in medicine. Implicit bias contributes to healthcare disparities. The film is focused on a middle-class Black woman's experience and was informed by a number of Black women's experiences, as well as research with Black and Latino/a/x patients. It is not meant to represent all Black women's experience or experience of all people of color. Likewise, it is not meant to represent all White doctors' experience. In addition to working with women of color for the patient perspective, this film was also informed by the experiences of several physicians, including those who practice in internal medicine, surgery, OB, pediatrics, and as GI subspecialists. Over 50 medical and creative professionals, patients, students and researchers were involved in the creation of the film.

This lesson was adapted from a 90-minute Facilitator's Guide written in partnership with NYU Grossman School of Medicine's Office of Diversity Affairs, The Empathy Project, Narrative 4, and Einstein College of Medicine and informed by the scholarship of Dr. Jodi Halpern, Dr. Jamil Zaki, and Dr. Cristina Gonzalez.

The Lesson

Implicit bias is part of human nature and our unique biases are developed through our lived experience, social norms, and the system in which we are socialized. Individuals can be biased on any number of constructs, including, but not limited to, race, gender, age, sexual orientation, religion, ethnicity, ability, and education. Problems or errors can occur when we don't recognize when implicit bias is influencing our interpersonal encounters. This 1-hour session will begin to show us what behaviors and skills we can use to recognize, manage, and address implicit bias in interpersonal encounters.

Materials

- "The Elephant in the Waiting Room" film: TEITWR_LC_MPEG.mp4

2022 New York University, all rights reserved. This material is available without charge for use for educational purposes only. No user is permitted to charge a fee for or otherwise profit by its use. New York University reserves the right to prohibit uses which in its sole judgment it deems inappropriate.

The Empathy Project uses the following definitions to ground the conversation:
Bias is prejudice in favor of or against one thing, person, or group compared with another, usually in a way considered to be unfair.

Implicit bias refers to unconscious and unintentional mental associations that impact our understanding and actions. Implicit bias recognition and management is a behavioral, skills-based approach to addressing implicit bias in interpersonal encounters.[1]

Empathy is the practice of authentically trying to understand another person's lived experience through communication, and, more specifically, through engaged curiosity.

Objectives:
- Recognize the impact of an individual's lived experience on the clinical encounter
- Identify behaviors perceived as aligned with implicit bias
- Use empathy by practicing perspective-getting and perspective-believing
- Develop strategies to recognize and mitigate implicit bias in clinical settings

Start the film and STOP at 02:20 when Ms. Ouologuem leaves her bathroom
QUESTIONS:
1. How do you think history or past experiences shape the characters' biases?
    - ❖ For Dr. Gunderson, reflect on his father's comment about the neighborhood being "bad." Biases are created by cultural norms and stereotypes shared between people and also through policies, such as redlining and racial segregation, that shaped neighborhoods. Also consider Dr. Gunderson's "color blind" medical training and the current day racial reckoning's impact on academic medicine.
    - ❖ For Ms. Ouologuem, reflect on how previous treatment experiences may have created trauma and informed Ms. Ouologuem's perception of health care. Consider the power differentials she's felt or the power she and her mother have been denied by society or an individual.
2. Consider how Ms. Ouologuem, in great pain, prepares for a visit to the clinic. What do you see in that scene? What do you learn about her and why she might go to such lengths to look and sound a certain way for the visit?

Resume and STOP FILM at 04:05
QUESTIONS:
1. How would you describe the interaction between the clinician and the patient up to this juncture in the movie?

[1] Cristina M. Gonzalez, Monica L. Lypson & Javeed Sukhera (2021) Twelve tips for teaching implicit bias recognition and management, Medical Teacher, 43:12, 1368-1373, DOI: 10.1080/0142159X.2021.1879378

2. What clinician behaviors impacted Ms. Ouologuem's perception of the encounter?
    - ❖ Asking where the patient is from based on a last name the doctor cannot pronounce
    - ❖ Looking at the computer while the patient is speaking
    - ❖ Standing up instead of sitting down
    - ❖ Glossing over the fact that she mentioned she is a software designer, etc.
3. What could Dr. Gunderson have done differently?
    - ❖ Developing the skill of perspective-getting can support clinicians in understanding when bias may be perceived by a patient they are caring for. Questions could be asked with engaged curiosity to gain (or get) another's perspective directly rather than from inferences. Developing and practicing this empathetic muscle is an important skill to support clinicians in building rapport with patients which will improve patient outcomes.

ADDITIONAL DISCUSSION FOR ADVANCED LEARNERS:

Think of a moment when you felt you, or a team member, were not acting with empathy or were not receiving empathy. What do you wish had happened instead? What could have been done to improve the clinical outcomes?

Resume the film and play it to the end:

QUESTIONS:

1. How are empathy and perspective-getting *and* believing important clinical skills for mitigating bias?
    - ❖ Perspective-getting and believing the patient allows clinicians to hear if bias may have been perceived.
2. Why is listening not enough? What is Denise trying to get across to Dr. Gunderson about perspective-getting and empathy when she says "You have to try and imagine how the world sees her and how she assumes the world sees her." – a core component of empathy.
    - ❖ Taking time to reflect cannot be overstated. It helps to develop empathetic imagination**,** an important skill to support clinicians in building or restoring rapport with patients and improving patient outcomes.
3. What role does Dr. Gunderson's apology play in restoring a rapport with Ms. Ouologuem?
    - ❖ Acknowledgement of real or perceived bias is shown to lead to outcomes similar to if the bias had never happened or been perceived in the first place. Acknowledgement or an apology is the most important act in restoring rapport with the patient. Ignoring or dismissing bias could lead to negative outcomes.

4. Compare and contrast verbal and nonverbal behaviors from before and after Denise the Empathy Elephant coaches Dr. Gunderson. Highlight the physical and emotional changes that have taken place in this scene.

TAKEAWAYS:

- A patient's lived experience may lead to perceived bias in a clinical encounter.
- Acknowledging one's own real or perceived bias is a strategy within implicit bias recognition and management to restore rapport.
- Practicing perspective-getting and believing are empathy skills which can contribute to the equitable outcomes we are all striving to achieve.

Even though Dr. Gunderson is a good doctor with good intentions, he did not employ perspective-getting and believing until Denise The Empathy Elephant coached him. By the end of the film Dr. Gunderson began to understand that whether it is perceived or actual bias, that the effect is the same for the patient. He learns the skill of empathy and that a sincere apology restores rapport with a patient once bias has been perceived.

EVALUATION/FEEDBACK

As part of the lesson, we ask every learner to complete the brief feedback form so we can continuously refine and improve The Empathy Project: https://nyumc.qualtrics.com/jfe/form/SV_cA9fqjpMLB5ew2G

If you are sharing the Feedback Form by PPT, please feel free to use the QR code below:
